# PGC-1α Silencing Compounds the Perturbation of Mitochondrial Function Caused by Mutant SOD1 in Skeletal Muscle of ALS Mouse Model

**DOI:** 10.3389/fnagi.2015.00204

**Published:** 2015-10-20

**Authors:** Yan Qi, Xiang Yin, Shuyu Wang, Hongquan Jiang, Xudong Wang, Ming Ren, Xiang-ping Su, Shi Lei, Honglin Feng

**Affiliations:** ^1^Department of Neurology, The First Clinical College of Harbin Medical University, Harbin, China; ^2^Department of Neurology, Uniformed Services University of the Health Sciences, Bethesda, MD, USA; ^3^College of Biological and Pharmaceutical Sciences, China Three Gorges University, Yichang, China

**Keywords:** ALS, SOD1(G93A), PGC-1α, energy metabolism, inflammation, fibrosis

## Abstract

Amyotrophic lateral sclerosis (ALS) is a lethal neurodegenerative disease causing death of motor neurons. This study investigated the roles of energy metabolism in the pathogenesis of ALS in the SOD1(G93A) transgenic mouse model. Control and SOD1(G93A) mice were administered with shcontrol or shPGC-1α in combination with PBS or thiazolidinedione (TZD) for 8 weeks. Gene expression was analyzed by quantitative real-time PCR and Western blot. ROS and fibrosis were assessed with a colorimetric kit and Sirius staining, respectively. Inflammatory cytokines were measured using ELISA kits. The levels of tissue ROS and serum inflammatory cytokines were significantly higher in SOD1(G93A) mice compared to control mice, and knocking down peroxisome proliferator-activated receptor gamma coactivator 1-alpha (PGC-1α) drastically increased cytokine levels in both control and SOD1(G93A) mice. Muscle fibrosis was much severer in SOD1(G93A) mice, and worsened by silencing PGC-1α and attenuated by TZD. The expression levels of PGC-1α, SOD1, UCP2, and cytochrome C were substantially reduced by shPGC-1α and increased by TZD in muscle of both control and SOD1(G93A) mice, whereas the level of NF-κB was significantly elevated in SOD1(G93A) mice, which was further increased by PGC-1α silencing. These data indicated that disruption of energy homeostasis would exacerbate the pathological changes caused by SOD1 mutations to promote the pathogenesis of ALS.

## Introduction

Amyotrophic lateral sclerosis (ALS), commonly known as Lou Gehrig’s disease in the USA, is a fatal neurodegenerative disease affecting motor neurons (Kiernan et al., 2011; Gordon, 2013). Although the cause of most ALS cases is not known, more genetic defects associated with familial ALS have been detected employing new technologies since the discovery of mutations in superoxide dismutase 1 (SOD1) (Rosen et al., 1993). Combining large-scale DNA sequencing with genome-wide association studies and linkage analysis, mutations in C9orf72 (DeJesus-Hernandez et al., 2011; Daoud et al., 2012; Robberecht and Philips, 2013), TDP43 (Sreedharan et al., 2008), and many other genes (Sreedharan and Brown, 2013) have been linked to or implicated in ALS. Among all the ALS-implicated genes, only SOD1 transgenic rodent models of ALS have been widely investigated (Gurney et al., 1994; Ripps et al., 1995; Dunlop et al., 2003).

The roles of mitochondrial dysfunction in the pathogenesis of ALS have been recognized since the identification of structural and morphological abnormalities in mitochondria from post-mortem skeletal muscle, liver, spinal cord neurons, and motor cortex of ALS patients (Hirano et al., 1984; Sasaki and Iwata, 1996, 1999). The defects in the activities of the electron transport complexes emerged during the pre-symptomatic phase of disease in the spinal cord of mutant SOD1 mice (Jung et al., 2002; Mattiazzi et al., 2002; Kirkinezos et al., 2005; Vehviläinen et al., 2014). Moreover, the impairments of mitochondria Ca^2+^ capacity and oxidative phosphorylation precede the onset of ALS (Kong and Xu, 1998; Damiano et al., 2006; Grosskreutz et al., 2010; Tan et al., 2014; Vehviläinen et al., 2014).

Peroxisome proliferator-activated receptor gamma coactivator 1-alpha (PGC-1α) is a transcriptional coactivator that regulates a wide range of genes involved in mitochondrial biogenesis, fatty acid oxidation, and oxidative metabolism (Wu et al., 1999; Liang and Ward, 2006), which has been implicated in Huntington’s disease, Parkinson’s diseases, ALS, and other neurodegenerative diseases (Róna-Vörös and Weydt, 2010). PPARγ agonists thiazolidinediones (TZDs) were shown to reduce hyperglycemia-induced ROS production and promoted mitochondria biogenesis through activating PGC-1α pathway (Fujisawa et al., 2009). Although the death of motor neurons is the most prominent characteristics of ALS, the cytotoxicity of SOD1(G93A) caused skeletal muscle atrophy might be an intrinsic characteristics of ALS pathogenesis (Dobrowolny et al., 2008). This study aimed to investigate the effect of manipulating PGC-1α pathway (activating PGC-1α by rosiglitazone or silencing PGC-1α) on the skeletal muscle of SOD1(G93A) ALS mouse model.

## Materials and Methods

### SOD1-G93A and C57BL/6 Mice

Four-week-old SOD1(G93A) mice (body weight about 75 g) were purchased from the Nanjing biomedical research institute. C57BL/6 mice were purchased from Yangzhou University. All animal protocols were reviewed and approved by the Institutional Animal Care and Usage Committee of The First Clinical College of Harbin Medical University. The mice were kept in a temperature-controlled (22–24°C) room with a 12-h light and 12-h dark cycle and allowed to customize to the new environment for a week. According to the different groups (10 mice in each group), injected PBS or rosiglitazone (GlaxoSmithKline, Philadelphia, PA, USA) (10 mg/kg body weight) with siControl or siPGC-1α through tail-vein twice a week for 8 weeks.

### Lentiviral shRNA Targeting PGC-1α

The shRNA targeting 5′-GGTGGATTGAAGTGGTGTAGA-3’ within mouse PGC-1α coding sequence (Koo et al., 2004) was designed and cloned into pLL3.7 lentiviral vector (MIT) according to the provider’s protocol. Lentiviruses (shPGC-1α and shControl) were packaged, purified, and tittered by syngentech (Beijing, China).

### Sirius Staining

Mouse gastrocnemius muscle tissue sections were stained with a Picric acid – Sirius staining kit (Senbeijia Biotech, Nanjing, China). The sections were washed three times in 1× PBS for 2 min each, incubated with Sirius staining solution at room temperature for 30 min, and washed as before, counterstained with hematoxylin for 5 min, washed three times in PBS for 1–2 min each before being checked and photographed under an Olympus ix71 microscope.

### Western Blot

The total proteins from gastrocnemius muscle were separated on a 12% SDS-polyacrylamide gel, transferred onto polyvinylidene difluoride membranes (Bio-Rad, Hercules, CA, USA). The membranes were blocked with 5% non-fat milk for 30 min at room temperature before being incubated with anti-PGC-1α (ab191838, Abcam, Cambridge, MA, USA), PGC-1β (ab199228, Abcam), SOD1 (ab52950, Abcam), UCP2 (ab77363, Abcam), NF-κB (ab194786, Abcam), P38 (ab119916, Abcam), Cytochrome C (ab133504, Abcam), or β-actin (ab194592, Abcam) antibodies overnight at 4°C. After washing, the membranes were incubated with horseradish peroxidase-conjugated secondary antibodies (Jackson ImmunoResearch Lab, West Grove, PA, USA) for 1 h at room temperature. The blots were then visualized using the enhanced chemiluminescence kit (Pierce, Rockford, IL, USA). Densitometry analysis was performed with a Hewlett-Packard scanner and NIH Image software (Image J).

### Quantitative Real-Time Polymerase Chain Reaction

Total RNA was extracted from mouse gastrocnemius muscle using RNeasy mini kits (Qiagen, Venlo, Netherlands). Reverse transcription was performed with the SuperScript^®^ III First-Strand Synthesis System (Life Tech, Shanghai, China) according to the supplier’s instructions using 1 g total RNA. Quantitative real-time PCR was performed using the SYBR^®^ Green PCR Master Mix (Life Tech) on a ABI 7300 (Applied Biosystems, Foster City, CA, USA) with the following program: 95°C for 3 min followed by 40 cycles of 95°C for 30 s, 58°C for 15 s, and 68°C for 30 s. The primers used were ACAGAGACACTGGACAGTCT and CATTGTAGCTGAGCTGAGTG for PGC-1α, CCTCCTTCTTCCTCAACTAT and GTTGGGTTCAGTCTCTGAGT for PGC-1β, ACTTCGAGCAGAAGGCAAGC and AACATGCCTCTCTTCATCCG for SOD1, CTTCACTTCTGCCTTCGGG and CCAGGATCCCAAGCGGAGA for UCP2, TGGGAAACCGTATGAGCCTG and GCAGAGTTGTAGCCTCGTGT for NF-κB, GTTCAGAAGTGTGCCCAGTG and TACTCCATCAGGGTATCCTC for cytochrome C, CCAGCTTAGGTTCATCAGGTAA and ACACCGACCTTCACCATTTTG for GAPDH. The relative transcription levels were calculated with the 2^−ΔΔCt^ method using GAPDH as the internal control.

### Immunoprecipitation

Mouse gastrocnemius muscle was washed twice with ice cold PBS and homogenized in 500 μl RIPA lysis buffer followed by three rounds of freeze-thaw. Tissue lysates were centrifuged for 5 min at 14,000 rpm at 4°C, and cleared supernatant was transferred into a new tube. For immunoprecipitations, 750 μg total protein was incubated with 5 μg anti NF-κB p65 (ab16502, Abcam), p50 (ab7971, Abcam) antibodies, or normal rabbit IgG (negative control), respectively, at 4°C overnight with rotation. Immune complexes were captured with 25 μl of protein A/G resin (Pierce) and incubated for 1 h at 4°C with rotation. Samples were washed four times with lysis buffer with centrifugation for 5 min at 2000 rpm at 4°C. After final wash, the pellets were boiled 5 min in 40 μl of 2× SDS sample buffer and subjected to SDS-PAGE and Western blot analysis.

### Cytokine Levels Measured by ELISA

The serum TNF-α, IL-1β, IL6, TNF-α, and MIP-1a levels were measured with specific ELISA kits (Elabscience, Wuhan, China) according to the manufacturer’s instructions.

### Measurement of Muscle ATP Level

Muscle ATP level was measured with a kit from Jianchen Biotech (A095, Nanjing, China) according to the manufacturer’s protocol. Briefly, mouse gastrocnemius muscle was homogenized in cold PBS followed by three freeze-thaw cycles. Supernatant was used for assay after lysates were centrifuged for 10 min at 13,000 g at 4°C. Thirty microliters of supernatant was missed with 100 μl of Substrate Solution I, 200 μl Substrate Solution II, and 30 μl Accelerant. The standard, blank, and control were set up according to the protocol. The mixtures were incubated at 37°C for 30 min and then mixed with 50 μl of Precipitant before being centrifuged for 5 min at 4000 rpm. Three hundred microliters of supernatant was mixed with 500 μl of color developing solution, incubated for 2 min at room temperature, stopped with 500 Stop Solution, and incubated for 5 min at room temperature before read at 636 nm. The ATP level was calculated as

$$\begin{array}{llll} \text{ATP}\left(\mu \text{ mol}/\text{g protein}\right) & =\left(\text{O}{\text{D}}_{\text{s}}-\text{O}{\text{D}}_{\text{c}}\right)/\left(\text{O}{\text{D}}_{\text{sd}}-\text{O}{\text{D}}_{\text{b}}\right)\; & & \;\\ & \;\times \text{1}0^{\text{3}}\mu \text{mol}/\text{L}\times \text{dilution}\; & & \;\\ & \;\times \text{Con}{\text{c}}_{\text{s}}\left(\text{g protein}/\text{L}\right)\; & & \; \end{array}$$

where OD_s_ was OD value of the sample, OD_c_ was OD value of control, OD_sd_ was OD value of standard, OD_b_ was OD value of blank, dilution was the fold sample was diluted before assay, and Conc_s_ was the concentration of the sample. The relative ATP level was calculated against wild-type mice treated with shControl and PBS.

### Analysis of the ROS Level in Mouse Skeletal Muscle

Muscle ROS level was measured with a commercial kit (E004, Jianchen Biotech) according to the manufacturer’s instruction. Briefly, mouse gastrocnemius muscle was homogenized in cold phosphate buffer (100 mmol/L, pH 7.1), centrifuged at 1000 g for 10 min at 4°C. One hundred ninety microliters of supernatant was mixed with 10 μl of freshly made 1 mmol/L DCFH-DC solution and incubated at 37°C for 30 min before measured at 500/525 nm (Ex/Em). The relative ROS levels were presented with the ROS level (OD525/mg protein) of wild-type mice that received shcontrol virus and PBS being set as 1.

### Statistical Analysis

The data was expressed as mean ± SE. The differences between groups were analyzed by one-way Analysis of Variance using Graphpad Prism 5. A *p*-value <0.05 was considered statistically significant.

## Results

### Silencing PGC-1α Potentiates ROS Production in SOD1(G93A) Mice

**Figure 1:**
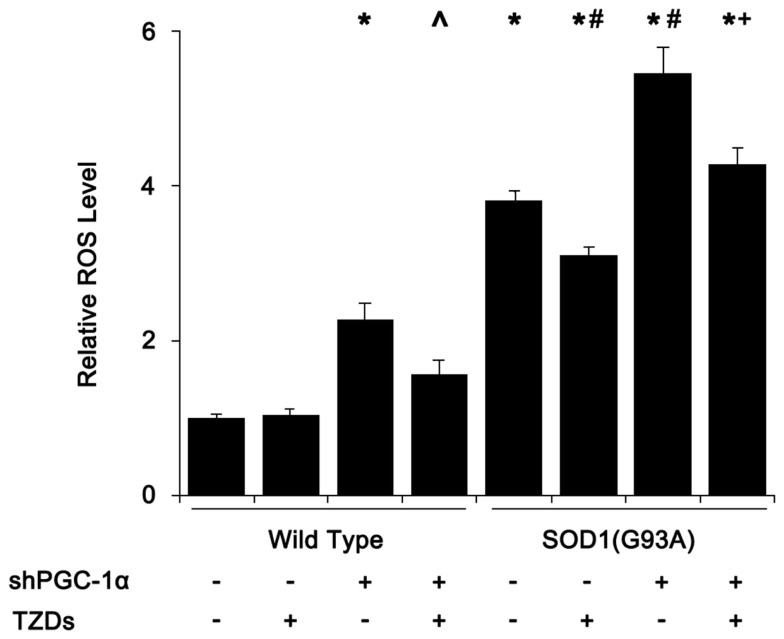
**SOD1(G93A) and PGC-1α silencing increased ROS levels in skeletal muscle**. Mouse gastrocnemius muscle was homogenized in 100 mmol/L phosphate buffer (pH 7.1) and centrifuged at 1000 g for 10 min. The supernatant (190 μl) was mixed with 10 μl of freshly made 1 mmol/L DCFH-DA working solution and incubated for 30 min at 37°C before measured at 500/525 nm (Ex/Em). Relative ROS levels were calculated with ROS level of wild-type mice that received shcontrol and PBS being set as 1. Data were expressed as mean ± SD (*n* = 10). *p < 0.05 vs wild-type mice treated with shControl and PBS; ^#^*p* < 0.05 vs SOD1(G93A) mice treated with shControl and PBS; ^∧^*p* < 0.05 vs wild-type mice treated with shPGC-1α and PBS; and ^+^*p* < 0.05 vs SOD1(G93A) mice treated with shPGC-1α and PBS.

As mitochondrial dysfunction and degeneration played a heavy part in the onset of ALS in SOD1(G93A) mice, we first assessed tissue ROS levels of mouse skeletal muscle. The ROS level of SOD1(G93A) transgenic mice was almost fourfold of that of wild-type mice, which was further increased to about 5.5-fold when PGC-1α was silenced in SOD1(G93A) transgenic mice (Figure 1). Silencing PGC-1α in WT mice resulted in more than doubled ROS level in muscle tissue (Figure 1). Treating SOD1(G93A) transgenic and/or PGC-1α knockdown mice with rosiglitazone reduced the ROS levels by 20–40%, respectively (Figure 1).

### PGC-1α Functions as a Inhibitor of Inflammation in SOD1(G93A) Mice

**Figure 2:**
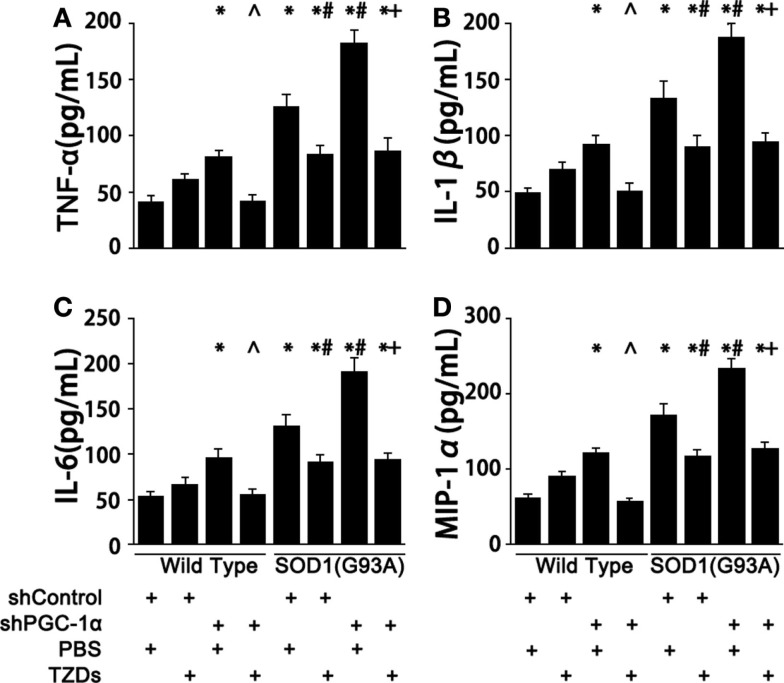
**PGC-1α knockdown exacerbated inflammation in SOD1(G93A) mice**. Mouse serum TNFα **(A)**, IL-1β **(B)**, IL-6 **(C)**, and MIP-1α **(D)** levels were measured with commercial ELISA kits. Data were expressed as mean ± SD (*n* = 10). *p < 0.05 vs wild-type mice treated with shControl and PBS; ^#^*p* < 0.05 vs SOD1(G93A) mice treated with shControl and PBS; ^∧^*p* < 0.05 vs wild-type mice treated with shPGC-1α and PBS; and ^+^*p* < 0.05 vs SOD1(G93A) mice treated with shPGC-1α and PBS.

Next, we examined the levels of inflammatory cytokines in those genetically manipulated mice since chronic-elevated ROS level initiated inflammation. Silencing PGC-1α in WT mice resulted in 70–100% increase of serum TNF-α, IL-1β, IL-6, and MIP-1α levels (Figure 2). The levels of proinflammatory cytokines of SOD1(G93A) mice were 2.4- to 3-fold of those of wild-type mice (Figure 2), which were increased about 40% more when PGC-1α was silenced in SOD1(G93A) mice (Figure 2). The increases of serum proinflammatory cytokines were inhibited by rosiglitazone treatment in those genetically manipulated mice (Figure 2).

### PGC-1α Silencing Exacerbates Muscular Fibrosis in SOD1(G93A) Mice

**Figure 3:**
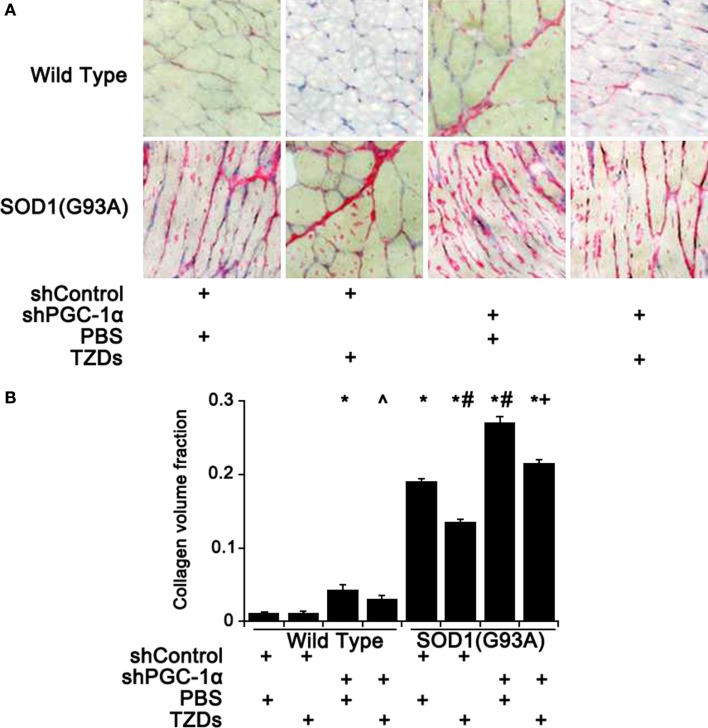
**Muscular fibrosis was increased by PGC-1α knockdown and SOD1(G93A)**. **(A)** The representative pictures of Sirius staining of gastrocnemius muscle from wild-type or SOD1(G93A) mice received different treatment. **(B)** Quantitative analysis of collagen volume fraction of mouse gastrocnemius muscles. Data were expressed as mean ± SD (*n* = 10). *p < 0.05 vs wild-type mice treated with shControl and PBS; ^#^*p* < 0.05 vs SOD1(G93A) mice treated with shControl and PBS; ^∧^*p* < 0.05 vs wild-type mice treated with shPGC-1α and PBS; and ^+^*p* < 0.05 vs SOD1(G93A) mice treated with shPGC-1α and PBS.

As muscular fibrosis was a major pathological feature of ALS, the fibrosis level of mouse gastrocnemius muscle was assessed by Sirius staining. Knockdown PGC-1α in wild-type mice resulted in about threefold increase of fibrosis in skeletal muscles compared to WT mice, which was attenuated by TZD treatment (Figure 3). The muscular fibrosis of SOD1(G93A) mice was more than 16.5 times of that of WT mice and further increased more than 40% with PGC-1α silencing (Figure 3). Treatment of rosiglitazone alleviated muscular fibrosis in all mutant mice (*p* < 0.01) even though it did not have any effect in WT mice (Figure 3).

### Muscular ATP Levels are Reduced by SOD1(G93A) Transgene and/or PGC-1α Knockdown

**Figure 4:**
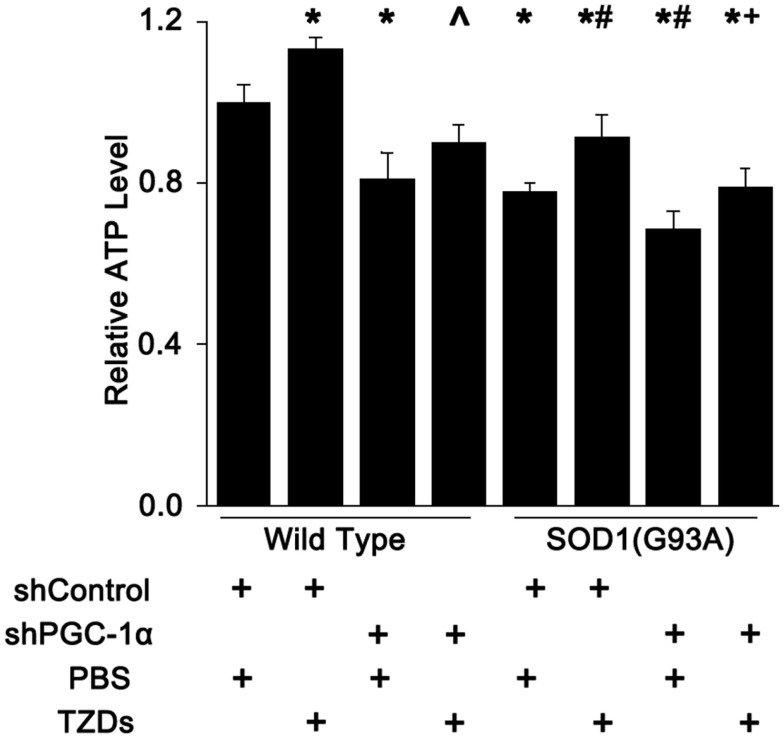
**Muscle ATP level was reduced by PGC-1α knockdown and SOD1(G93A) transgene**. The ATP levels in mouse gastrocnemius muscle were measured with a commercial colorimetric kit and expressed as levels relative to wild-type mice received shcontrol and PBS. Data were expressed as mean ± SD (*n* = 10). *p < 0.05 vs wild-type mice treated with shControl and PBS; ^#^*p* < 0.05 vs SOD1(G93A) mice treated with shControl and PBS; ^∧^*p* < 0.05 vs wild-type mice treated with shPGC-1α and PBS; and ^+^*p* < 0.05 vs OD1(G93A) mice treated with shPGC-1α and PBS.

Next, we checked the ATP levels mouse gastrocnemius muscle since PGC-1α and SOD1(G93A) were shown to regulate the number or function of mitochondria. The muscular ATP levels were reduced about 20% in SOD1(G93A) transgenic mice and PGC-1α knockdown mice, whereas it was decreased about another 10% in mice harboring both SOD1(G93A) transgene and PGC-1α knockdown (Figure 4). Rosiglitazone treatment increased muscular ATP levels by more than 10% in mice with all genotypes tested (Figure 4).

### PGC-1α and SOD1(G93A) Regulate Genes Involved in Metabolism, Antioxidant, and Inflammation

**Figure 5:**
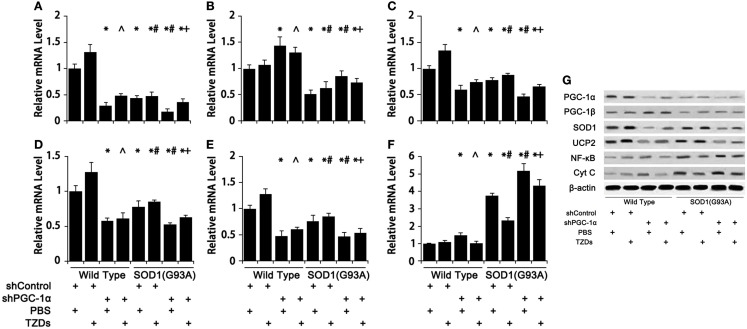
**The expression of genes related to energy metabolism, oxidative stress, and inflammation was changed in mice with SOD1(G93A) transgene and/or PGC-1α knockdown**. The mRNA levels of PGC-1α **(A)**, PGC-1β **(B)**, SOD1 **(C)**, UCP2 **(D)**, Cyt C **(E)**, and NF-κB **(F)** were analyzed with real-time qPCR and protein levels **(G)** with Western blot. Data were expressed as mean ± SD (*n* = 10). *p < 0.05 vs wild-type mice treated with shControl and PBS; ^#^*p* < 0.05 vs SOD1(G93A) mice treated with shControl and PBS; ^∧^*p* < 0.05 vs wild-type mice treated with shPGC-1α and PBS; and ^+^*p* < 0.05 vs SOD1(G93A) mice treated with shPGC-1α and PBS.

The expression pattern of genes involved in energy metabolism and inflammation in mouse gastrocnemius muscle were assessed to evaluate the effects of PGC-1α silencing in combination with SOD1(G93A) transgenic on these physiological processes. Silencing PGC-1α drastically down-regulated SOD1 (Figure 5C), UCP2 (Figure 5D), and cytochrome C (Figure 5E) expression. On the other hand, SOD1(G93A) transgenic significantly reduced the mRNA levels of PGC-1α (Figure 5A), PGC-1β (Figure 5B), endogenous SOD1 (Figure 5C), UCP2 (Figure 5D), and cytochrome C (Figure 5E), whereas markedly increased NF-κB mRNA level (Figure 5F) was in gastrocnemius muscle compared to WT mice. Knockdown PGC-1α further strengthened those changes, while TZD treatment showed a trend of partially counteracting those changes (Figure 5). The protein levels of aforementioned genes were generally consistent with their corresponding mRNA levels (Figure 5G).

### Decreased PGC-1α Results in Increased Free NF-κB

**Figure 6:**
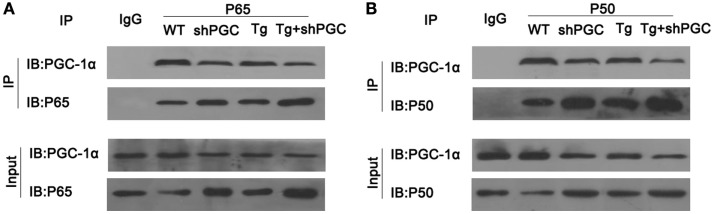
**PGC-1α physically interacted with NF-κB**. The total protein of mouse gastrocnemius muscle was immunoprecipitated with antibody against NF-κB p65, NF-κB p50, or IgG and immunoblotted with specified antibodies. PGC-1α was co-precipitated with NF-κB complex by antibodies against NF-κB p65 **(A)** and p50 **(B)** but not by control IgG. Tg, SOD1(G93A) transgenic; shPGC, shPGC-1α.

As inflammatory responses and the injuries caused by inflammation played a critical part in ALS pathogenesis, we tested if PGC-1α was directly involved in the regulation of inflammation. After immunoprecipitation of total protein of mouse gastrocnemius muscles using antibodies against NF-κB subunits p65 and p50, PGC-1α was detected in the immunocomplexes of NF-κB, whereas it was not precipitated by IgG control (Figure 6). Moreover, compared to WT mice, the amount of precipitated PGC-1α from SOD1(G93A) and/or shPGC-1α mice was significantly decreased by antibodies against either NF-κB subunits p65 or p50 even though the amount of p65 and p50 precipitated from the mutant mouse samples was higher than that of WT mouse (Figure 6).

## Discussion

SOD1(G93A) transgene and PGC-1α knockdown individually or additively inhibited the expression of genes involved in oxidative metabolism (cytochrome C), antioxidant (SOD1), mitochondria biogenesis (PGC-1α and PGC-1β), and uncoupling (UCP2) but up-regulated inflammation gene (NF-κB) in mouse gastrocnemius muscles, which led to reduced muscular ATP levels, elevated ROS level, increased inflammation and muscular fibrosis.

The onset and progression of ALS involved many pathological changes other than the death of motor neurons. The activation of microglia and the progression of non-autonomous death of motoneurons were significantly slowed down by reducing mutant SOD1 in astrocytes (Yamanaka et al., 2008) and microglia (Boillée et al., 2006) of SOD1(G37R) mice. Deletion of the tumor necrosis factor-like weak inducer of apoptosis (Tweak) in SOD1(G93A) transgenic mice significantly reduced microgliosis and inflammation, and ameliorated ALS-associated pathological changes in skeletal muscles (Bowerman et al., 2015). Skeletal muscle atrophy and denervation were shown to precede the loss of motor axons from the ventral root and the death of motor neurons (Fischer et al., 2004). Moreover, as one of the most energy consuming tissues, skeletal muscle is essential for maintaining the metabolic homeostasis (Palamiuc et al., 2015) and is the primary target of the cytotoxicity of mutant SOD1 (Dobrowolny et al., 2008; Wong and Martin, 2010). In SOD1(G86R) transgenic mice, blocking muscular metabolism switch from glucose to lipids by inhibiting pyruvate dehydrogenase kinase 4 activity with dichloroacetate increased PGC-1α level, reduced oxidative stress, and ultimately improved the disease conditions (Palamiuc et al., 2015). Mice with muscle specific SOD1(G37R) transgenic progressively developed muscular atrophy with myofiber loss and muscle cell apoptosis, microgliosis, neuromuscular junction abnormalities, motoneuron distal axonopathy, and eventually death of motor neurons (Wong and Martin, 2010), indicating that ALS might originate from pathogenesis of skeletal muscle.

Muscular hypermetabolism, weight loss, fat mass reduction, and switching energy production from glycolysis to lipid consumption were presented in ALS patients and ALS mouse models (Dupuis et al., 2004, 2011; Dorst et al., 2011; Palamiuc et al., 2015). Increased serum lipid levels showed a protective effect in ALS patients (Dupuis et al., 2008; Dorst et al., 2011) and SOD1(G93A) mice (Dupuis et al., 2004), which might be due to providing increased energy sources. These changes might be resulted from the perturbation of the link between PPARγ-regulated lipid metabolism and mitochondrial oxidative phosphorylation (Capitanio et al., 2012), suggesting that effective ALS intervention may be required to coordinate lipid oxidation with the increase of mitochondrial number (PGC-1α) and the improvement of the integrity and efficiency of mitochondria (Liu et al., 2011).

**Figure 7:**
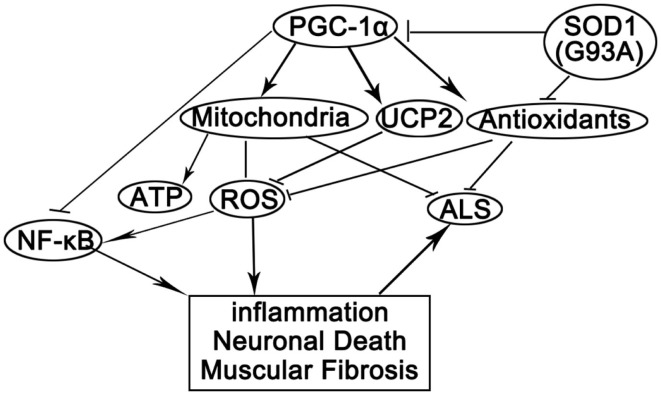
**A proposed model for the actions of PGC-1α and SOD1(G93A) in ALS pathogenesis**. PGC-1α orchestrates mitochondria biogenesis and antioxidant enzymes while inhibits NF-κB activity. Knockdown PGC-1α causes ATP deficiency and elevated oxidative stress. SOD1(G93A) inhibits PGC-1α pathway and antioxidants among others. Reduced ATP level, reduced antioxidants, and increased oxidative stress cause inflammation, neuron degeneration, and myopathy, which promotes the progression of ALS.

In this study, we showed that PGC-1α silencing in SOD1(G93A) mice compounded with mutant SOD1 to cause increased oxidative stress, inflammation, energy deficiency, and massive muscular fibrosis. These pathological changes could be attenuated by peroxisome proliferator-activated receptor gamma (PPARγ) agonist TZDs. By summarizing our data and previously reported results, we postulated that mutant SOD1 and/or PGC-1α silencing might have several effects on the skeletal muscles (Figure 7). First, SOD1(G93A) transgene and PGC-1α knockdown caused the perturbation of metabolism and energy homeostasis due to mitochondrial deficiency and abnormal oxidative metabolism (Wu et al., 1999; Liang and Ward, 2006; Róna-Vörös and Weydt, 2010; Palamiuc et al., 2015). Second, oxidative stress was increased due to the reduced expression of antioxidant enzymes and uncoupling proteins as well as mitochondrial malfunction (Wu et al., 1999; Liu et al., 2002; Róna-Vörös and Weydt, 2010) in SOD1(G93A) transgenic and/or PGC-1α knockdown mice. Third, inflammatory responses towards ROS and other stimuli were augmented since NF-κB was released from the inhibitory complex with PGC-1α due to the reduction of PGC-1α level (Wang et al., 2007).

In conclusion, mitochondrial dysfunction and metabolism imbalance were at the center of ALS pathogenesis. Mutant SOD1 decreased PGC-1 expression and mitochondrial biogenesis, resulted in energy deficiency, increased ROS, caused massive inflammation and muscle fibrosis. Knockdown PGC-1α exacerbated aforementioned changes. Strategies simultaneously increasing mitochondria number, mitochondrial integrity, and lipid metabolism may be explored for ALS treatment.

## Conflict of Interest Statement

The authors declare that the research was conducted in the absence of any commercial or financial relationships that could be construed as a potential conflict of interest.
