# Corrigendum: PGC-1α silencing compounds the perturbation of mitochondrial function caused by mutant SOD1 in skeletal muscle of ALS mouse model

**DOI:** 10.3389/fnagi.2016.00037

**Published:** 2016-03-01

**Authors:** Yan Qi, Xiang Yin, Shuyu Wang, Hongquan Jiang, Xudong Wang, Ming Ren, Honglin Feng

**Affiliations:** ^1^Department of Neurology, The First Clinical College of Harbin Medical UniversityHarbin, China; ^2^Department of Neurology, The Affiliated Hospital of Weifang Medical UniversityWeifang, China

**Keywords:** ALS, SOD1(G93A), PGC-1α, energy metabolism, inflammation, fibrosis

Due to the author's misunderstanding of the Frontiers platform and the criteria for author contributions, Xiang-ping Su and Shi Lei were mistakenly included as authors on the Original Research article “PGC-1α silencing compounds the perturbation of mitochondrial function caused by mutant SOD1 in skeletal muscle of ALS mouse model.”

We, the authors, received technical assistance from Xiang-ping Su and Shi Lei during the process of submission, review and production, and the general use of the Frontiers system, but Xiang-ping Su and Shi Lei had no contribution to the research submitted. As such they should not be considered as authors on this article. Both Xiang-ping Su and Shi Lei agree to the removal.

The authors apologize for this error and misunderstanding.

In addition, due to the author's oversight, some minor typographical errors were included in the Materials and Methods section:

In sub-section SOD1-G93A and C57BL/6 Mice, the body weight of 4-week-old SOD1(G93A) mice was reported as “about 75 g.” The correct body weight was about 15 g.

In sub-section Quantitative Real-Time Polymerase Chain Reaction, the reverse transcription was reported as performed “using 1 g total RNA.” The correct unit was 1 μg total RNA.

In sub-section Immunoprecipitation, the protein incubation was reported as “750 μg.” The correct total protein incubated was 75 μg.

The authors apologize for these errors and any confusion caused.

These errors do not change the scientific conclusions of the article in any way.

## Author contributions

YQ: experiments design and participation, materials purchase, data collection and analysis, paper writing; YX: experiments design and participation, data collection and analysis, paper writing; SW: experiments design and participation, animal raising, specimens collection; HJ: experiments design and participation, materials purchase, technical support; XW: experiments design and participation, data collection and analysis, paper writing; MR: experimental instruction and participation, paper writing; FF: experiments design, instruction and participation, paper writing. All authors meet all four of the below criteria: (1) Substantial contributions to the conception or design of the work; or the acquisition, analysis, or interpretation of data for the work; (2) Drafting the work or revising it critically for important intellectual content; (3) Final approval of the version to be published; (4) Agreement to be accountable for all aspects of the work in ensuring that questions related to the accuracy or integrity of any part of the work are appropriately investigated and resolved.

## Funding

This research was supported in part by the grants from the Natural Science Foundation of China (No. 81171186).

### Conflict of interest statement

The authors declare that the research was conducted in the absence of any commercial or financial relationships that could be construed as a potential conflict of interest.

